# Impact of CDK4/6 Inhibitors on Aromatase Inhibitor-Associated Musculoskeletal Syndrome (AIMSS) in the Adjuvant Setting

**DOI:** 10.1155/2023/3614296

**Published:** 2023-05-31

**Authors:** Efthymia Skafida, Angeliki Andrikopoulou, Evangelos Terpos, Christos Markellos, Savvina Moustafa, Dimitrios Pectasides, Meletios-Athanasios Dimopoulos, Flora Zagouri, Dimitrios Vassilopoulos

**Affiliations:** ^1^Department of Clinical Therapeutics, Alexandra Hospital, Medical School, Athens, Greece; ^2^Clinical Immunology-Rheumatology Unit, 2nd Department of Medicine and Laboratory, National and Kapodistrian University of Athens School of Medicine, Hippokration General Hospital, Athens, Greece; ^3^Oncology Section, Second Department of Internal Medicine, Hippokration Hospital, Athens, Greece

## Abstract

**Background:**

Third-generation aromatase inhibitors (AIs) are the mainstay of treatment in hormone receptor (HR)-positive breast cancer. Even though it is considered to be a well-tolerated therapy, AI-induced musculoskeletal symptoms are common and may be accused for treatment discontinuation. Recently, selective cyclin-dependent kinase 4 and 6 (CDK4/6) inhibitors changed the therapeutic setting, and currently, ribociclib, palbociclib, and abemaciclib are all approved in combination with nonsteroidal AIs in patients with ER-positive, HER2-negative advanced or metastatic breast cancer. This systematic review aims to identify the frequency of aromatase inhibitor-associated musculoskeletal syndrome (AIMSS) in the adjuvant setting in patients under AI monotherapy compared to patients under combination therapy with AIs and CDK4/6 inhibitors and demonstrate the underlying mechanism of action.

**Methods:**

This study was performed in accordance with PRISMA guidelines. The literature search and data extraction from all randomized clinical trials (RCTs) were done by two independent investigators. Eligible articles were identified by a search of MEDLINE and ClinicalTrial.gov database concerning the period 2000/01/01–2021/05/01.

**Results:**

Arthralgia was reported in 13.2 to 68.7% of patients receiving AIs for early-stage breast cancer, while arthralgia induced by CDK4/6 inhibitors occurred in a much lower rate [20.5–41.2%]. Bone pain (5–28.7% vs. 2.2–17.2%), back pain (2–13.4% vs. 8–11.2%), and arthritis (3.6–33.6% vs. 0.32%) were reported less frequently in patients receiving the combination of CDK4/6 inhibitors with ET.

**Conclusions:**

CDK4/6 inhibitors might have a protective effect against joint inflammation and arthralgia occurrence. Further studies are warranted to investigate arthralgia incidence in this population.

## 1. Introduction

Breast cancer is the most common malignancy in females affecting more than 2,000,000 women each year [1]. Clinical practice has changed substantially in recent decades leading to a more prolonged survival. At the time of diagnosis, approximately 64% of patients have localized disease, while 6% of patients have already developed metastases. The 5-year survival rate of metastatic disease is estimated to be around 27% [2]. Around 75% of all breast tumors express the estrogen receptor (ER) and/or the progesterone receptor (PgR) and are considered as hormone receptor-positive (HR) tumors [3]. HR-positive breast cancer demonstrates the best survival rates (90.3%–92.5% 4-year survival rate) compared with epidermal growth factor receptor 2 (HER2)-positive tumors (82.7% 4-year survival rate) and triple-negative subtype (survival rate 77.0%) [4].

Adjuvant endocrine therapy remains the mainstay of HR-positive early breast cancer along with chemotherapy and adjuvant radiation therapy. For postmenopausal women, next generation aromatase inhibitors (AIs) nonsteroidal (anastrozole and letrozole) or steroidal (exemestane) are widely used in the adjuvant setting [5–7]. However, administration of AIs has been associated with joint pain and musculoskeletal symptoms that can even lead to treatment discontinuation. Aromatase inhibitor-associated musculoskeletal syndrome (AIMSS) is the most common adverse event encountered by breast cancer patients. In early-stage breast cancer, an incidence of 47% of arthralgias was reported in postmenopausal women treated with AI, including a 23.5% rate of new-onset arthralgia and a 23.5% rate of exacerbation of pre-existing arthralgias [8]. Another recent meta-analysis showed a 17.9% rate of AI-induced arthralgia in postmenopausal women which was lower in early-stage breast cancer compared with advanced disease [9]. Overall, approximately 13–25% of patients discontinue AI therapy mainly because of arthralgias [10, 11].

The development of selective cyclin-dependent kinase 4 and 6 (CDK4/6) inhibitors which regulate cell cycle progression has altered daily clinical practice. Currently, ribociblib (LEE011), palbociclib (PD0332991), and abemaciclib (LY2835219) are all approved in combination with nonsteroidal AIs as first-line therapy of ER-positive, HER2-negative advanced or metastatic breast cancer. Results from Phase III studies showed a 40–45% improvement in progression-free survival (PFS) from the addition of CDK4/6 inhibitors in the metastatic setting [12]. CDK4/6 inhibitors are currently under investigation in the adjuvant setting as well. Adverse events commonly reported after CDK4/6 inhibitor administration includes neutropenia (75–80%) upon ribociclib or palbociclib treatment, elevated amino-transferases (15%), and prolongation of corrected QT interval by Fredericia (QTcF) (3.3%) with ribociclib and diarrhea (81.3%) upon abemaciclib treatment. Remarkably, musculoskeletal symptoms have not been linked with CDK4/6 treatment despite the coadministration with AIs. Whether CDK4/6 inhibitors affect the arthralgia rate reported in Phase III trials of AIs remains unknown. We conducted this systematic review to identify the rate of musculoskeletal manifestations induced by AIs and CDK4/6 inhibitors in the adjuvant setting and the potential effect of CDK4/6 inhibitors on joint symptoms. We have previously published the musculoskeletal toxicity of CDK4/6 and aromatase inhibitors in the metastatic setting, so we are now presenting the existing data in the adjuvant setting [13].

## 2. Materials and Methods

This systematic review was performed in accordance with PRISMA guidelines [14]. The protocol of this systematic review has been submitted to the Institutional Review Board of Alexandra Hospital and Medical School of Athens and is available upon request. All eligible studies were identified using MEDLINE and ClinicalTrial.gov database for search concerning the period 2000/01/01–2021/05/01. The search algorithm applied consisted of the following words: (breast AND (cancer OR neoplasm) and (aromatase inhibitors OR letrozole OR anastrozole OR exemestane OR CDK4/6 OR palbociclib OR ribociclib OR abemaciclib) AND (phase III) AND (adjuvant). Language restrictions were not applied. In order to maximize the amount of synthesized information, we systematically examined the reference lists of the articles retrieved for potentially eligible studies.

Eligible studies included all randomized controlled Phase III trials exploring third-generation AI monotherapy treatment in early-stage breast cancer in postmenopausal women and all randomized controlled Phase III trials evaluating AIs in combination with CDK4/6 inhibitors in the adjuvant setting. Phase I-II trials, case reports, and reviews were excluded. Trials of AIs in combination with other antineoplastic drugs, e.g., trastuzumab, or in combination with bone-protective therapies [15–19] were also excluded. In case of overlapping publications emerging from the same study, the larger sample size study was evaluated, e.g., TEAM trial and N-SAS BC 04 trial [6, 20]. In case of additional information provided from multiple papers from the same trial, each article was evaluated separately [5, 21].

From each of the eligible studies, the following data were extracted: first author, year of publication, trial number, treatment arms, sample size, median age, median follow-up time, disease-free survival, overall survival, arthralgia rate, myalgia rate, backpain rate, bone pain rate, arthritis rate, osteoporosis, and osteoporotic fracture rates. Two investigators (ES and AA), working independently, searched the literature, and extracted data from each eligible study. Only data when AIs were administered as monotherapy treatment were considered eligible, and thus, data emerging from the sequential administration of tamoxifen and AIs were not included in our final results. In addition, whenever study results were available on Clinical.Trials.gov, results extracted from eligible articles were updated according to Clinical.Trials.gov data. Any differences in extracted data were resolved via within-pair consensus. Odds Ratio (OR) with 95% confidence interval (CI) was applied as an effect estimate of arthralgia occurrence when CDK4/6 inhibitors were added to ET therapy. Pooled OR with 95% CI was calculated using the random-effects model due to the underlying variations across the included trials. Heterogeneity across included trials was assessed using *I*^2^ and *Q* statistics, and significant heterogeneity was defined as *I*^2^ > 50.0% or *P* < 0.10. Statistical analysis was conducted using Review Manager (RevMan) v.5.4.1 software.

## 3. Results

**Figure 1:**
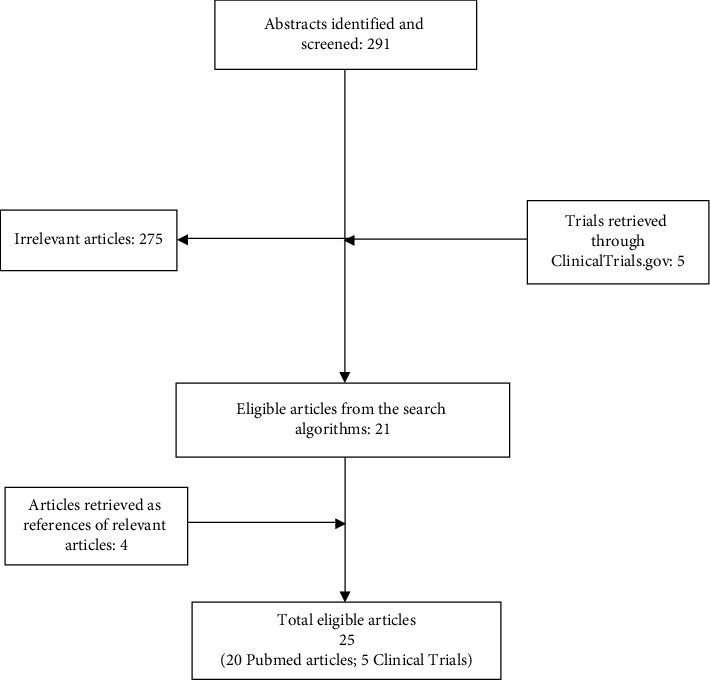
Flowchart presenting the successive steps during the selection of studies.

Overall, 291 articles were identified and screened in the MEDLINE database. Among them, 16 studies were considered eligible for our review [5, 6, 21–38]. After investigating the references of the eligible articles, four more studies were added (ITA [39], ARNO-95 [40, 41], ABCSG-6a [42], NSABP B-33 [43]). An additional search in ClinicalTrials.gov recruited five additional studies (PENELOPE-B[44], ATAC [7, 45], IES [46], GIM4 LEAD [47], SALSA [48]). Overall, 25 studies were considered as eligible for this systematic review. The flow diagram of literature search and study selection is shown in Figure 1. There are no data about whether the manifestations of AIMSS were defined according to a specific protocol or by investigator's judgment in each trial.

### 3.1. Arthralgia

In the adjuvant setting, the reported percentage of arthralgias in patients under monotherapy with AIs ranged from 13.2 to 68.7%, while in patients receiving therapy with tamoxifen, the reported arthralgia rates ranged from 11.8 to 31.8% and in patients receiving placebo from 15 to 50% (Table 1). The highest rates of arthralgias were observed in studies when 4–6 years of AI treatment had preceded before enrolment, such as MA.17R study and SOLE study where arthralgia was reported in 53% and 68.7% of patients, respectively [32, 36]. Anastrozole and letrozole were associated with approximately the same arthralgia rate (48.2% vs. 47.9%, respectively) according to the FACE trial [28]. More specifically, letrozole monotherapy induced arthralgia with an incidence of 13.2–68.7%, while the incidence of arthralgia in anastrozole and exemestane monotherapy was 24.5–57.7% and 24–55.4% respectively. Overall, the occurrence of exemestane-induced arthralgia is lower than those of letrozole and anastrozole.

**Figure 2:**
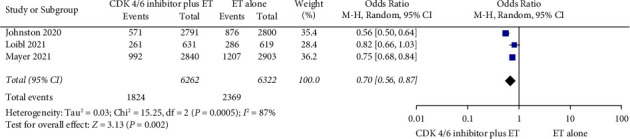
Forest plot of the arthralgia incidence in patients receiving CDK4/6 inhibitors plus ET versus ET alone.

Data from MonarchE, PALLAS, and PENELOPE-B Phase III trials of CDK4/6 inhibitors plus endocrine therapy (ET) versus ET in the adjuvant setting were just published [35, 37, 38, 44] (Table 2). Arthralgia rate was 20.5–41.2% of patients receiving CDK4/6 inhibitor plus ET. In all of the trials, arthralgia rate was lower in patients receiving CDK4/6 inhibitors compared to the control arm (MonarchE: 20.5% vs. 31.3%; PALLAS: 34.9% vs. 41.6%; PENELOPE-B: 41.2% vs. 46.8%). The odds ratio of arthralgia incidence ranged from 0.56 to 0.82 across these trials. Pooled analysis of these trials showed that adding CDK4/6 inhibitor to ET significantly reduced arthralgia incidence (OR: 0.70; 95% CI: 0.56–0.87; *P* < 0.05; Figure 2) compared to ET alone despite the heterogeneity observed across the trials (*I*^2^ = 87%; *P* < 0.05). Of note, arthralgia incidence was lower in abemaciclib treatment (20.5%) compared to palbociclib (34.9–41.2%) [35, 37, 38, 44].

### 3.2. Myalgias

In the adjuvant setting, the incidence of myalgia reported in 9 of RCT was 7.5–37.1% in patients receiving AIs as monotherapy, 7% in patients receiving tamoxifen, and 6–25% in patients receiving placebo. The highest myalgia rates were reported in MA.17R trial (28%) and SOLE trial (35.9%/37.1%) where patients enrolled had received 4 to 5 years of previous endocrine therapy with AIs [32, 36]. In the rest of RCTs, the myalgia rate reported was lower than 20% (7.5–17.7%). Myalgia incidence was 8–37.1%, 10.3–16.7%, and 7.5–17.7% in early breast cancer patients treated with letrozole, anastrozole, and exemestane, respectively. There are no data of myopathy or elevated CPK in any of the trials.

Myalgia incidence was reported in only two of the trials of CDK4/6 inhibitors in the adjuvant setting [38, 44]. Myalgia rate ranged from 5.98 to 20.2% in patients treated with CDK4/6 inhibitors plus ET for early breast cancer which was comparable to myalgia rates reported in the control arms (MonarchE: 5.98% vs. 5.92%; PENELOPE-B: 20.2% vs. 18.5%).

### 3.3. Bone Pain

Data concerning bone pain emerge from 9 RCTs and affect 5–28.7% of patients receiving AIs in the adjuvant setting, while 6–14% and 16% of patients suffered from bone pain in the placebo and tamoxifen groups, respectively. The incidence of bone pain in exemestane group was 5.4–10.1%, whereas it was 5–28.7% and 5.9–10.9% in the letrozole and anastrozole groups.

Bone pain was reported by 2.2–17.2% of patients receiving CDK4/6 inhibitors [38, 44]. In accordance with arthralgia rate, the incidence of bone pain was also lower in patients receiving the combination of CDK4/6 inhibitors with ET compared to ET alone (MonarchE: 2.2% vs. 3.3%; PENELOPE-B: 17.2% vs. 19.1%).

### 3.4. Backpain

The percentage of backpain observed in patients treated with AIs in the adjuvant setting was 2–13.4% according to reported data emerging from 5 RCTs. The incidence of backpain for letrozole was 2–10.3% compared with 9.4–13.4% and 5.7–12.7% reported in anastrozole and exemestane arms.

Backpain was reported in 8–11.2% of patients treated with abemaciclib or palbociclib [38, 44]. Backpain was less frequently identified in patients receiving CDK4/6 inhibitors compared to the control arms (MonarchE: 8% vs. 9.7; PENELOPE-B: 11.2% vs. 13.3%).

### 3.5. Arthritis

Arthritis is of major concern in patients treated with AIs, and data concerning arthritis were reported in 6 trials. In the adjuvant setting, AIs induced arthritis in 3.6–33.6% of women, which was higher than the 12% and 5–30% rates, reported in tamoxifen monotherapy and placebo groups, respectively. However, the rate of AI-induced arthritis was 6–7% in the majority of RCTs [6, 22–24] with the exception of MA.17R trial where AI treatment for 4.5–6 years preceded the enrolment (33%) [36]. The arthritis rate caused by letrozole was 6–33%, while arthritis was reported in 6.4% of women in anastrozole group and 3.6–7% in exemestane group.

Only one trial has reported the incidence of arthritis induced by CDK4/6 inhibitors in the adjuvant setting [38]. Arthritis was identified in 0.32% of patients receiving abemaciclib plus ET in the adjuvant setting.

### 3.6. Osteoporosis-Osteoporotic Fractures

Data concerning osteoporosis and osteoporotic fractures were eligible in 13 and 19 of the 25 RCTs, respectively. In breast cancer patients receiving letrozole, osteoporosis was confronted in 4.8–46.8% of patients. Osteoporosis incidence was 10.9–36.4% and 10–33% in patients receiving anastrozole and exemestane, respectively. The highest rates of osteoporosis were reported in SOLE trial where about half of patients treated with letrozole for five years after prior endocrine therapy for 4–6 years developed osteoporosis. Osteoporosis incidence increased consistently with duration of AI treatment according to results of DATA, IDEAL, and GIM4 LEAD trials [29, 31, 47]. CDK4/6 inhibitors (abemaciclib) plus ET induced osteoporosis less commonly (1.61%) than ET alone (4.8–46.8%) in MonarchE trial [38].

Fractures represent one of the serious adverse events in AI treatment. In the adjuvant setting, incidence of fractures observed in patients treated with AIs ranged from 0.5% to 14%. The highest fracture rate (14%) was reported in MA.17R trial where patients received AIs for a total of 10 years while the lowest rate (0.5%) was reported in GIM4 LEAD trial where patients received AI treatment for a maximum of 5 years after treatment with tamoxifen for 2 to 3 years [36, 47]. In contrast, incidence of fracture was 1–7.7% and 1.1–9% in patients treated with tamoxifen or placebo, respectively. The incidence of fracture was greater in patients treated with AIs compared with tamoxifen treatment as shown in BIG 1–98, ATAC, TEAM, IES, ABCSG-6a, and ABCSG-8/ARNO-95 trials [5–7, 21, 22, 40, 42, 45, 46]. The duration of AI treatment seemed to correlate with fracture rate. DATA trial reported an 7.5% and 10% fracture rate in patients treated with anastrozole for 3 and 6 years, respectively [29]. Consistently, patients administered with letrozole for 2.5 and 5 years presented with fractures in a rate of 2.8% and 5% respectively in IDEAL trial [31]. SALSA trial demonstrated an increase from 4% to 6% of fracture rate in patients receiving anastrozole for 2 versus 5 years, respectively [48, 49]. Overall, letrozole induced fractures in 0.5–14% of patients, whereas the same rates were 0.8–11% and 4–5% for anastrozole and exemestane population.

### 3.7. Carpal Tunnel Syndrome

Carpal tunnel syndrome was reported in 0–3% of patients treated with AIs in the adjuvant setting in contrast with 0.2–1% incidence reported in tamoxifen arms according to data reported in 4 RCTs. There are no data of the incidence of carpal tunnel syndrome in patients receiving CDK4/6 inhibitors in the adjuvant setting.

## 4. Discussion

Aromatase inhibitor-induced musculoskeletal syndrome (AIMSS) has emerged as a major cause of treatment discontinuation in hormone receptor-positive patients treated with AIs. In our systematic review of phase III RCTs, arthralgias were reported in 13.2 to 68.7% of patients receiving AIs for early-stage breast cancer, while arthralgias induced by CDK4/6 inhibitors occurred in a much lower rate [20.5–41.2%]. Pooled analysis of three trials evaluating CDK4/6 inhibitors plus ET in the adjuvant setting demonstrated an odds ratio of arthralgia in favor of the population receiving CDK4/6 inhibitors (OR: 0.70; 95% CI: 0.56–0.87; *P* < 0.05) although the populations included were quite heterogeneous. Bone pain (5–28.7% vs. 2.2–17.2%), back pain (2–13.4% vs. 8–11.2%), and arthritis (3.6–33.6% vs. 0.32%) were reported less frequently in patients receiving the combination of CDK4/6 inhibitors with ET. Our results are consistent with data emerging from previous studies. Crew et al. reported a 47% rate of AI-related joint pain and 44% rate of joint stiffness in postmenopausal women with early-stage breast cancer [8]. Consistently, 45% of women with early-stage breast cancers experienced joint symptoms according to results from COBRA trial [50]. Median time to onset of symptoms was 1.6 months although joint symptoms could appear as early as a few days after initiation of treatment [50]. Laroche et al. reported an initial appearance of joint pain after 6 weeks of treatment and a consequent more diffuse pain after 12 months of treatment [51]. Whatever the time course, AI-induced musculoskeletal syndrome remains of major clinical significance in women with breast cancer.

AIs may lead to joint symptoms via three different mechanisms. Estrogen deprivation seems to be the most likely mechanism of AIMSS [52]. Treatment with AIs results in a decrease in serum estrogen levels below postmenopausal levels. The expression of the two estrogen receptors alpha and beta (ER*α* and ER*β*) in human articular cartilage provides evidence that estrogens modulate the metabolism of chondrocytes [53]. Concentrations of ER*α* and ER*β* receptors are increased in men, and thus, male joints may be more protected against the development of arthritis [53]. Animal studies demonstrated that estrogen deficiency may accelerate cartilage turnover and increase cartilage surface erosion [54]. Exogenous estrogen or SERM administration suppresses the progression of cartilage erosion exerting a chondroprotective effect on articular cartilage. Moreover, hormone replacement therapy results in a reduction up to three-fold of osteoarthritis incidence [55, 56]. In addition, estrogen has direct effects on opioid pain fibers in the central nervous system [57]. Estrogen receptors are localized in opioid-containing neurons in the spinal cord and brain. Apart from their role in central nervous system, estrogens enhance neural transmission of peripheral nociceptive input by inducing an inflammatory environment within the joint. As a result, transmission of stimuli produced from articular structures is facilitated via estrogen mediation.

The second mechanism of AI-induced arthralgia might implicate an autoimmune process [52]. Morel et al. described a case of rheumatoid arthritis in a woman treated with exemestane [58]. Moreover, AI treatment may lead to autoimmune diseases in patients with breast cancer, including rheumatoid arthritis, subacute cutaneous lupus erythematosus (SCLE), and vasculitis [59]. This could be due to AI-induced inhibition of differentiation of naïve T-cells to regulatory T-cells and increased interferon-*γ* (IFN-*γ*) and interleukin-12 (IL-12) cytokine levels. In some cases, autoimmune disorders including arthralgias reversed upon AI cessation, along with a decrease in autoantibody levels, such as antinuclear antibodies (ANA) and rheumatoid factor (RF) [60]. The last and less likely mechanism of AI-induced musculoskeletal syndrome is through a direct off target effect of the AI medication or one of its metabolites [52].

CDK4/6 inhibitors have been widely applied in everyday clinical practice in patients with HR-positive disease. Cyclin-dependent kinase 4 or 6 (CDK4/6) modulates cell cycle progression to the S phase via catalyzing retinoblastoma (RB) protein hyperphosphorylation [12]. In the hypophosphorylated state, RB exerts a repressive effect on the E2F family of transcription factors blocking cell cycle progression through the G1-S checkpoint. In response to mitogenic signals, including ER pathway, cyclin D associates with the protein kinases CDK4 and CDK6, and the cyclin D-CDK4/6 complex subsequently phosphorylates the RB protein. Phosphorylation induces structural changes in RB protein rendering it unable to interact with E2F transcription factors. CDK4/6 inhibitors restore the onco-suppressive effect of RB by preventing its hyperphosphorylation by CDK4/6 kinases.

The mechanism through which CDK4/6 inhibitors might reduce the rate of AIMSS remains unclear. Handschick et al. demonstrated that CDK6 physically and functionally interact with NF-kB subunit p65 [61]. Aberrant CDK6 expression contributes to NF-kB p65 phosphorylation and NF-kB-induced gene expression. NF-kB is activated upon stimulation from proinflammatory cytokines, such as interleukin-1 (IL-1) or tumor necrosis factor alpha (TNF-a). It was shown that 34–45% of IL-1-induced genes required CDK4 or CDK6, while RNAi-mediated knockdown of CDK6 suppressed several IL-1-induced genes such as IL-8, IL-6, and NFKB1A [61]. Moreover, CDK6 colocalized with NF-kB p65 subunit at several binding sites modulating the expression of many NF-kB target genes. CDK4/6 inhibitors might attenuate CDK6-dependent inflammatory gene expression and the development of an intraarticular inflammatory microenvironment. The inhibition of AI-induced inflammation by CDK4/6 inhibitors could contribute to the decreased arthralgia rate observed in this population.

CDK4/6 inhibitors reinstate the suppressive effect of RB on the E2F family of transcription factors. Wang et al. demonstrated that E2F2 regulates the inflammation produced via STAT1 and PI3K/AKT/NF-kB pathways in a rheumatoid arthritis animal model [62]. Indeed, NF-kB associates with the promoter region of E2F2, and activated E2F2 subsequently binds to the promoter of IL-6, which in turn leads to the development of arthritis. SiRNA-mediated knockdown of E2F2 attenuates the expression of inflammatory cytokines in rheumatoid arthritis synovial fibroblasts. E2F2 regulates nuclear translocation of STAT1 and activation of PI3K/AKT/NF-kB pathway, inhibiting the expression of inflammatory cytokines such as IL-1 and TNF-a. This effect of E2F2 is further supported by the fact that E2F2 is overexpressed in RA synovial tissues [62]. CDK4/6 inhibitors might suppress E2F2 through an RB-dependent way and thus inhibit the hyperplasia of RA synovial fibroblasts and joint damage caused by E2F2-induced inflammatory factors. Tomita et al. reported a decrease in cartilage erosion by infiltrating synovium upon transfection with E2F decoy oligodeoxynucleotides (ODN) [63]. The production of IL-1, IL-6, and MMP-1 proinflammatory factors was also impaired by E2F decoy ODN. CDK4/6 inhibitors might have a similar protective function against articular cartilage invasion.

## 5. Conclusion

Overall, we report a decreased incidence of AI-induced arthralgia in postmenopausal breast cancer patients treated with AI and CDK4/6 inhibitor combination in the adjuvant setting. CDK4/6 inhibitors might have a protective effect against joint inflammation and arthralgia occurrence although the exact mechanism remains unknown. Ongoing Phase III trials of CDK4/6 inhibitors in the adjuvant setting remain to address this issue.

## Figures and Tables

**Table 1:** Phase III randomized trials of AIs in postmenopausal women with early-stage breast cancer.

Reference	Trial	Treatment arms	Study sample	Median age	Median follow-up (month)	DFS	OS	Arthralgia (%)	Myalgia (%)	Bone pain (%)	Back pain (%)	Arthritis (%)	Osteoporosis (%)	Fracture (%)	Carpal tunnel syndrome
Colleoni et al. [21]	BIG 1–98 (NCT00004205)	Tamoxifen 5 years vs. letrozole 5 years	4922 (2459/2463)	61.7/61.9	51	81.1%/84% IPCW: 82.1/85.6	90.4/91.8	16.6/22.5	7/8.4	NR	NR	NR	2.2/5.1	6.7/10	0.2/0.8
Regan et al. [5]															

Buzdar et al. [45]	ATAC (NCT00849030)	Tamoxifen for 5 years vs. anastrozole for 5 years vs. combination for 5 years	9366 (3116/3125/3125)	64.1/64.1/64.3	68	575 vs. 651 events; HR: 0·87	Similar; HR: 0.97	29.4/35.6	NR	NR	NR	NR	7/11	7.7/11	1/3
Howell et al. [7]															

Van de Velde et al. [6]	TEAM (NCT00036270, NCT00279448)	Tamoxifen for 2.5–3 years plus exemestane until 5 years vs. exemestane for 5 years	9779 (4875/4904)	64	61	5-year: 86%/87%; HR: 0.93	5-year: 91%/91%; HR: 1.0	20.9/24	7.4/7.5	4.8/5.4	5.7/5.7	2.9/3.6	6/10	3/5	NR
						10-year: 67%/67%; HR: 0.96	10-year: 73%/74%; HR: 0.98								
Derks et al. [22]															

Coombes et al. [46]	IES (NCT00038467)	Tamoxifen for 2–3 years plus exemestane until 5 years vs. tamoxifen for 5 years	4724 (2352/2372)	63.7/63/6	55.7	5-year: 354 vs. 455 events; adjusted HR: 0.75	5-year: 222 vs. 261 deaths; adjusted HR: 0.85	18.6/11.8	NR	NR	NR	14.1/12	7.3/5.5	4.3/3.1	2.8/0.3

Goss et al. [23, 24]	NCIC CTG MA-17 (NCT00003140)	Letrozole vs. placebo for 5 years (after 5 years of tamoxifen)	5187 (2593/2594)	62.4/62	30	4-year: 94.4%/89.8%; HR:0.58	4-year: 95.4%/95%; HR: 0.82	25/21	15/12	5/6	NR	6/5	8.1/6	5.3/4.6	NR

Goss et al. [36]	MA.17R (NCT00754845)	Letrozole vs. placebo for 5 years (after 4.5–6 years of AI treatment preceded or not by tamoxifen)	1918 (959/959)	65.6/64.8	75	5-year: 96%/91%; HR: 0.66	5-year: 93%/94%; HR: 0.97	53/50	28/25	18/14	NR	33/30	11/6	14/9	NR

Mamounas et al. [25, 26]	NSABP-B42 (NCT00382070)	Letrozole vs. placebo for 5 years (after 5 years of tamoxifen or AI)	3966 (1983/1983)	NR	83	7-year: 84.7%/81.3%; HR: 0.85	7-year: 91.8%/92.3%; HR: 1.15	18/15	8/6	NR	2/2	NR	NR	3/2	NR

Smith, et al. [28]	FACE (NCT00248170)	Letrozole vs. anastrozole for 5 years	4136 (2061/2075)	62/62	65	5-year: 84.9%/82.9%; HR: 0.93	5-year: 89.9%/89.2%; HR: 0.98	48.2/47.9	11.4/10.3	6.7/5.9	10.3/9.4	NR	10.9/10.9	NR	0/0.05
Boccardo et al. [39]	ITA trial (NCT00286117)	Tamoxifen for 2–3 years plus anastrozole until 5 years vs. tamoxifen for 5 years	448 (223/225)	63/63	36	94.6%/85.8%	10 deaths vs. 4 deaths	NR	NR	NR	NR	NR	NR	1/1.3	NR

Jakesz et al. [42]	ABCSG 6a (NCT00300508)	Anastrozole for 3 years vs. placebo (after 5 years of tamoxifen)	856 (387/469)	67.8/68.5	62.3	5-year: 7.15 vs. 11.8%; 16 vs. 35 events (HR = 0.53; *P*=0.034)	5-year: 40 deaths (10.3%) vs. 55 deaths (11.7%); HR: 0.89; *P*=0.570	24.5/18.3	NR	NR	NR	NR	NR	0.8/1.1	NR

Dubsky et al. [27]	ABCSG-8 (NCT00291759)	Tamoxifen for 2 years plus anastrozole for 3 years vs. tamoxifen for 5 years	3714 (1865/1849)	63.6/64	60	5-year: 89.5%/88.5%; HR: 0.91	83 vs. 94 deaths; HR: 0.87	NR	NR	19/16	NR	NR	NR	2.3/1.5	NR
Jakesz et al. [40]															

Jakesz et al. [40]	ABCSG-8 (NCT00291759)	Tamoxifen for 2 years plus anastrozole for 3 years vs. tamoxifen for 5 years	3224 (1618/1606)	60.9/60.5	28	3-year: 95.8%/92.7%; HR: 0.60	3-year: 97%/96%; HR, 0.53	NR	NR	NR	NR	NR	NR	2/1	NR
	ARNO 95 (NCT00287534)														

Tjan-Heijnen et al. [29]	DATA (NCT00301457)	Anastrozole for 3 years vs. 6 years (after 2-3 years of tamoxifen)	1860 (929/931)	57.6/57.7	51	5-year: 79.4%/83.1%; HR: 0.79	5-year: 90.4%/90.8%; HR: 0.91	52.5/57.7	NR	NR	NR	NR	16.4/20.9	7.5/10	NR

Colleoni et al. [32]	SOLE (NCT00553410)	Letrozole intermittently or continuously for 5 years (after 4–6 years of endocrine therapy)	4851 (2425/2426)	60/60	60	5-year: 85.8%/87.5%; HR: 1.08	5-year: 94.3%/93.7%; HR:0.85	65.7/68.7	35.9/37.1	27.2/28.7	NR	NR	47.4/46.8	8.1/8.8	NR

Blok et al. [31]	IDEAL (BOOG 2006−05)	Letrozole for 2.5 years vs. 5 years (after 5 years of endocrine therapy)	1824 (909/915)		79	5-year: 82%/83.4%; HR: 0.92	HR: 1.04	13.2/14.7	NR	NR	6.1/5	NR	7.5/12.7	2.8/5	NR
De Placido et al. [30]	FATA-GIM3 (NCT00541086)	Anastrozole or exemestane or letrozole 5 years vs. tamoxifen for 2 years plus anastrozole or letrozole or exemestane for 3 years	3697 (1847/1850)	64/64	60	5-year: 89.8%/88.5%; HR: 0.89	5-year: 96.8%/95.3%; HR: 0.72	NR	17/13	27.3/21.9	NR	33.6/25.8	23.8/19.4	4/5	NR

Del Mastro et al. [47]	GIM4 LEAD (NCT01064635)	Letrozole for 2–3 years vs. 5 years (after 2–3 years of tamoxifen)	2056 (1030/1026)	60/61	120	8-year: 80%/85%; HR: 0.82	NR	NR	NR	NR	NR	NR	4.8/8.3	0.5/0.9	NR

NCT00295620 [48, 49]	SALSA (ABCSG 16)	Anastrozole for 2 years vs. 5 years (after 5 years of endocrine therapy)	3470 (1732/1738)	65/65		5-year: 78%/78%; HR 0.99	NR	NR	NR	NR	NR	NR	NR	4/6	NR

Mamounas et al. 2008 [43]	NSABP B-33 (NCT00016432)	Exemestane for 5 years vs. placebo for 5 years (after 5 years of tamoxifen)	1598 (799/799)		30	4-year: 91%/89%; *P*=0.07	16 vs. 13 deaths	1/0.5 (Grade3/4)	NR	0.5/0.7 (Grade 3/4)	NR	NR	NR	NR	NR

Aihara et al. [33]	N-SAS BC03	Tamoxifen for 1–4 years plus anastrozole until 5 years vs. tamoxifen for 5 years	706 (354/3520)	59.5/60.2	42	3-year: 94.3%/90.7% HR: 0.69	3-year: 99.6%/98.8%	50.4/31.8	NR	NR	NR	NR	NR	1.4/2.6	NR

Ruiz-Borrego et al. [34]	GEICAM/2006−10 (NCT00543127)	Anastrozole vs. anastrozole plus fulvestrant	870 (437/433)	62/62	75	7-year: 83.3%/86.9%; HR: 0.84	5-year: 95.8%/94.3%; *P*=0.558	14.6/13.4 (Grade 2–4)	2.7/5 (Grade 2–4)	3/6.5 (Grade 2–4)	NR	NR	NR	NR	NR

Goss et al. [23, 64]	NCIC CTG MA.27 (NCT00066573)	Exemestane for 5 years vs. anastrozole for 5 years	7576 (3,789/3,787)	64.1	49	4-year: 91 vs. 91.2; HR: 1.02	4-year: 94.5%/94.1; HR: 0.93	55.4/55.4	17.716.7	10.1/10.9	12.7/13.4	7/6.4	33/36.4	4/4	NR

NR = not reported.

**Table 2:** Phase III randomized trials of CDK4/6 inhibitors in postmenopausal women with early-stage breast cancer.

Reference	Trial	Treatment arms	Study sample	Median age	Median follow-up (month)	iDFS	OS	Arthralgia (%)	Myalgia (%)	Bone pain (%)	Back pain (%)	Arthritis (%)	Osteoporosis (%)	Fracture (%)	Carpal tunnel syndrome
Johnston et al. [35, 38]	MonarchE	Abemaciclib plus endocrine therapy (ET) vs. ET (AIs, tamoxifen)	5637 (2808/2829)	51/51	15.5	2-year: 92.2% vs. 88.7% *e* (*P*=0.01; HR: 0.75)	—	20.5%/31.3%	5.98%/5.92%	2.22%/3.32%	8%/9.7%	0.32%/1.04%	1.61%/2.54%	NR	NR

Mayer et al. [37]	PALLAS	Palbociclib plus endocrine therapy (ET) vs. ET (AIs, tamoxifen)	5760 (2883/2877)	52/52	23.7	3-year: 88.2% vs. 88.5% (*p*=0.51; HR: 0.93)	—	34.9%/41.6%	NR	NR	NR	NR	NR	NR	NR

Loibl et al. [44]	PENELOPE-B	Palbociclib plus endocrine therapy (ET) vs. ET (AIs, tamoxifen) after taxane-containing NACT	1250 (631/619)	49/48	42.8	3-year: 81.2% vs. 77.7% (HR: 0.93; *P*=0.525)	3-year: 93.6%/90.5% (HR: 0.87; *P*=0.420)	41.2%/46.8%	20.2%/18.5%	17.2%/19.1%	11.2%/13.3%	NR	NR	NR	NR

NR = not reported; iDFS: invasive disease-free survival; NACT: neoadjuvant chemotherapy.

## Data Availability

The data supporting our findings can be found in PubMed MEDLINE bibliographical and ClinicalTrial.gov database.
